# Seasonal Effects of Glucosinolate and Sugar Content Determine the Pungency of Small-Type (Altari) Radishes (*Raphanus sativus* L.)

**DOI:** 10.3390/plants11030312

**Published:** 2022-01-25

**Authors:** Seung-Hun Chae, O New Lee, Han Yong Park, Kang-Mo Ku

**Affiliations:** 1 BK21 Interdisciplinary Program in IT-Bio Convergence System, Chonnam National University, Gwangju 61186, Korea; kjcmc0921@naver.com; 2 Department of Horticulture, Chonnam National University, Gwangju 61186, Korea; 3 Department of Bioindustry and Bioresource Engineering, Sejong University, Seoul 05006, Korea; onewlee@sejong.ac.kr

**Keywords:** glucosinolate, pungent, radish, season, raphasatin

## Abstract

Kimchi made from small-type (Altari) radishes grown in late spring is more pungent than that made from autumn-grown Altari radishes, which poses a major challenge in the kimchi industry. The mechanism through which the pungency of Altari radish changes seasonally has not been intensively investigated. In this study, three small-type radish cultivars with different pungency levels were cultivated in spring and autumn to identify the factors affecting the seasonal-dependent pungency of small-type radishes. The contents of pungency-related metabolite glucoraphasatin and other polar metabolites were analyzed. Although a previous study reported that the glucoraphasatin concentration affects the pungency of radish, in the current study, the concentration of neither glucoraphasatin nor its hydrolysis product (raphasatin) could fully explain the change in the pungency associated with radish cultivars grown in the two seasons. The change in the pungency of radish by season may be explained by the ratio of raphasatin content to total sweetness of sugars. In addition, the polar metabolites that differ with season were analyzed to identify seasonal biomarkers and understand the seasonal changed physio-biochemistry.

## 1. Introduction

Radish (*Raphanus sativus* L., 2n = 18) is an important root vegetable belonging to the Brassicaceae family; it has been historically cultivated in China, India, Japan, Korea, European countries, and America [1]. In Korea, many radish F1 hybrid cultivars have been developed for seasonal cropping systems (Autumn, Winter, Spring, and Summer) and consumer tastes (big root, small root; Altari radish, young leaf; Yeolmu, and processing for pickling) [2]. Standardizing radishes is difficult due to their differences in taste depending on the season and cultivar; therefore, the taste of radish kimchi differs due to the characteristics of the constituent radishes [3]. The glucosinolate content in radish has a simple profile because it has low number of glucosinolates, despite the various morphological variations among the germplasms, such as root shape and weight [4].

Kimchi is a unique and traditional fermented food in Korea with a historical background of over 1000 years [5]; 467,000 tons of kimchi were produced in Korea in 2018 [6]. Kimchi is mainly prepared by fermenting kimchi cabbage or radish using lactic acid bacteria [7].

Glucosinolates, which are secondary sulfur-containing metabolites, are commonly found in the members of the *Brassica* species [8]. Three types of glucosinolates have been identified depending on their amino acid precursor: aliphatic glucosinolates, derived from methionine; aromatics, derived from tryptophan or phenylalanine; and indolic glucosinolates, derived from tryptophan [4,9], with aliphatic glucosinolates being the most common type in the *Brassica* crops. The glucosinolate content in *Brassica* vegetables is affected by environmental conditions, abiotic stresses, and genetic background [4]. The most abundant glucosinolate found in radish roots is 4-methylthio-3-butenyl glucosinolate (glucoraphasatin), also known as dehydroerucin, an aliphatic glucosinolate derived from methionine. More than 90% of total glucosinolate from Japanese and Chinese radish types were known to be glucoraphasatin [10]. Previous studies reported that glucoraphasatin concentration in radish root is significantly affected by different cultivars [11]. Previously, 4-methylthio-3-butenyl isothiocyanate (raphasatin) was isolated from radish root and identified as pungent [12]. It has been reported that radish has very low or non-epithiospecifier protein activity [13,14,15], resulting in an extremely high conversion rate from glucoraphasatin to raphasatin by endogenous myrosinase. Thus, the pungency of radish can be determined by its glucoraphasatin concentration. The concentration of raphasatin can be different due to myrosinase activity [16]. Myrosinase activity can be changed by seasonal or environmental conditions [17,18,19,20]. Therefore, to explain the pungency of radish, quantification of raphasatin is necessary. Glucosinolates are known to play a role in plant defenses against fungi, nematodes, herbivores, and weeds [21]. Isothiocyanates derived from the breakdown of glucosinolates in damaged tissue impart a pungent and bitter flavor and a sulfurous aroma to *Brassica* vegetables [8,22]. Taken together, previous results suggested that raphasatin concentration can be affected by biotic and abiotic factors.

Seasonal variations in glucosinolate content have been reported in several *Brassica* species, such as radish, turnip, cabbage, and oilseed rape [23,24,25,26,27]. A positive relationship between soil temperature and glucosinolate content was reported in *Brassica oleracea* [19]. Water stress caused by drought also increased the glucosinolate content in several *Brassica* species [28]. In addition, sugars impart distinctive sweetness, are a source of carbon and energy, and play a role as a signal molecule for developmental and metabolic processes in the plant life cycle [29]. High temperatures led to the prompt degradation of sucrose into glucose and fructose in radish [30]; however, the optimum temperature (22/18 °C) rapidly increased the sugar content in the hypocotyl in parallel with thickening root growth in radish [30]. The sweetness in the taste of radish is correlated with the presence of glucose and fructose [31].

Altari radish kimchi, also known as Chonggak kimchi, is popular in Korea. Dissatisfaction with the pungent flavor due to the presence of raphasatin is one of the most common complaints regarding Altari radish kimchi. Similarly, in Japan, the strong pungent taste of raw radish and its processed products (grated Oroshi radish) is considered to be the reason for lower consumer preference [14]. Although the pungency of radishes in general is decreased through the loss of myrosinase and glucosinolate in fermentation under acidic conditions [32], the pungency of late-spring-grown Altari radish kimchi made from spring- or summer-grown radish is greater than that made from autumn-grown Altari radish kimchi. Moreover, the effect of genotype on the glucoraphasatin content of radish root has been reported in several studies [19]. However, few studies have examined the effects of the growing season on the compound responsible for the pungent flavor radish (raphasatin) and other taste-related compounds in small-type radish roots. Thus, this study analyzed the glucosinolates and their hydrolysis products and other taste-related compounds in three small-type radish cultivars grown in two growing seasons (spring and autumn) in Korea [33]. In the current study, we applied a comprehensive metabolomic approach to identify potential pungency-determining compounds explaining differences between these three radish cultivars with different levels of pungency.

## 2. Results and Discussion

### 2.1. Growing Environmental Factors

**Figure 1:**
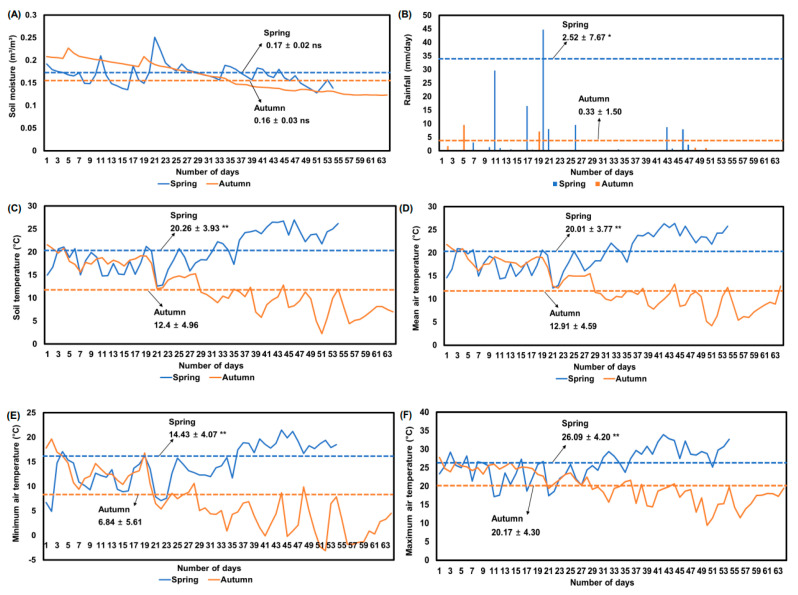
Seasonal average soil moisture (**A**); rainfall (**B**); soil temperature (**C**); mean air temperature (**D**); minimum air temperature (**E**); and maximum air temperature (**F**) during spring and autumn plantings for radish (*Raphanus sativus* L.) in 2020. Blue (spring) and orange (autumn) dashed line indicated average value of each environment condition. Ns and asterisks (* and **) next to mean and standard deviation values (dashed blue and orange line) indicate significant differences between spring and autumn by *t*-test at *p* > 0.05, *p* < 0.01, and *p* < 0.001, respectively.

The growing environmental factors used in this study are displayed in Figure 1. Soil temperature, rainfall, mean air temperature, minimum air temperature, and maximum air temperature in spring were significantly higher than those of autumn. The soil moisture of spring (0.17 m^3^/m^3^) was not significantly different from the soil moisture of autumn (0.16 m^3^/m^3^, Figure 1A). However, soil moisture of two weeks before harvesting was significantly higher in spring (0.16 m^3^/m^3^) than in autumn (0.14 m^3^/m^3^) by unpaired *t*-test (*p* < 0.05). In addition, rainfall was significantly higher in spring (2.52 mm/day) than in autumn (0.33 mm/day) by unpaired *t*-test (*p* < 0.05). Soil temperature was significantly higher in spring (20.3 °C) than in autumn (12.4 °C) by unpaired *t*-test (*p* < 0.05). After three weeks of sowing radish seeds in spring and autumn, air temperatures (mean, minimum, and maximum) and soil temperature were gradually increased or decreased, respectively, which was the most different one among growing environmental factors. The growing environmental conditions exhibited the typical spring–autumn differences of Korea.

### 2.2. Quantification of Glucosinolate

**Figure 2:**
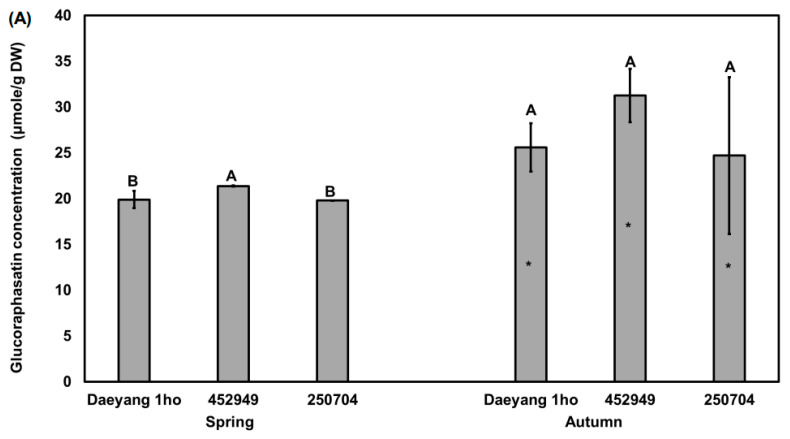

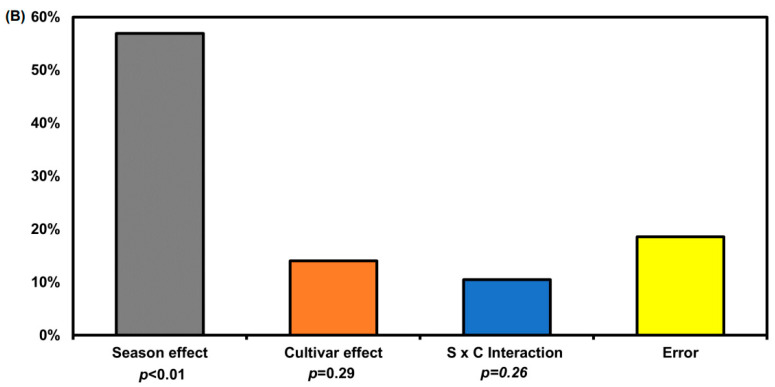
(**A**) Glucoraphasatin content in the roots of three different radish cultivars grown in spring and autumn 2020. Different capital letters above the error bars indicate significant differences among cultivars within the same season according to Tukey’s HSD test (*p* < 0.05). Asterisks (*) inside the bars indicate significant differences between spring and autumn within the same cultivar according to an unpaired Student’s *t*-test (*p* < 0.05); (**B**) Two-way ANOVA for glucoraphasatin content in radish roots of three different cultivars across two seasons. S × C indicates the interaction of season and cultivar. % indicated % of explained variation.

Glucoraphasatin from radish root accounted for more than 92% of glucosinolates from Japanese and Chinese radish cultivars [10]. In addition, Kim et al. [12] reported that the pungent odor of radish is related to the glucoraphasatin content, which is its major glucosinolate. Sonam et al. [34] also reported that total glucosinolate was significantly correlated with glucoraphasatin (*p <* 0.01). Thus, it is important to measure glucoraphasatin of radish root to explain its pungency. Glucosinolate content was significantly different between spring and autumn in each of the three cultivars by unpaired *t*-test (*p* < 0.05; Figure 2A), with the content in the spring root of cultivar ‘452949’ being significantly higher than that in other cultivars (*p* < 0.05). In autumn, the average glucoraphasatin content in the radish roots from three cultivars was 27.18 μmole/g DW, and 20.23 μmole/g DW in spring, which is about twice as high as that in autumn. As analyzed by the two-way analysis of variance (ANOVA) for season and cultivar, the changes in the glucoraphasatin content in the roots were also affected by the season (57%, *p* < 0.01, F = 18.38; Figure 2B) while glucoraphasatin content in the roots was not significantly affected by cultivar effect. From this analysis, we found that the difference in glucoraphasatin content amongst the three different cultivars was not significant in autumn season while spring season showed significant differences among the three cultivars. Apparently, the low temperature of the autumn growing season might have affected the glucosinolate biosynthesis of radishes in our study: the average soil- and air-temperatures in autumn were significantly lower than those in spring, respectively (Figure 1). We could not find any previous publication on glucoraphasatin concentration change in radish roots by temperature. A previous study using *Brassica oleracea* reported that the aliphatic glucosinolate content in the root was significantly decreased at 32 °C compared to 12 °C [19]. Average rainfall in autumn was significantly lower than that in spring, which would have affected glucoraphasatin content. Ciska et al. [35] reported that the aliphatic glucosinolate content of various *Brassica oleracea* crops and turnip varied significantly over two years with temperature and rainfall (*p* < 0.001). The high glucoraphasatin in autumn may be produced more by drought stress caused by soil moisture (Figure 1A) and high temperature (Figure 1C,D) during the harvest time. As a result of quantifying the four glucosinolates (progoitrin, glucoraphanin, glucoraphasatin, and neoglucobrassicin, Supplement Figure S1), concentration of three glucosinolates (progoitrin, glucoraphanin, and neoglucobrassicin) account for only 5% of total glucosinolates, which cannot explain pungency. Neoglucobrassicin, which is known to be bitter from broccoli and cabbage [36,37], was not significantly different by unpaired *t*-test. (Supplement Table S1). Although the difference in glucoraphasatin concentration between two season was significant, the glucoraphasatin difference does not fully explain the different levels of bitterness among cultivars that we experienced. This does not match the fact that spring radish is more bitter than autumn radish. Therefore, we analyzed the effect of hydrolysis product on bitter taste [36].

### 2.3. Glucosinolate Hydrolysis Products

**Figure 3:**
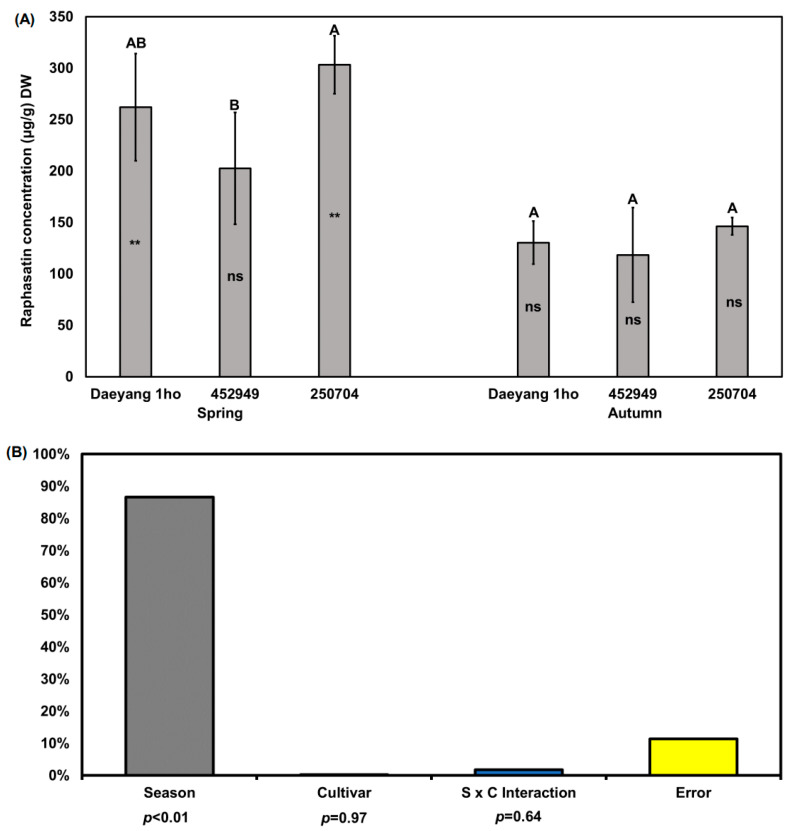
(**A**) Raphasatin concentrations in the roots of three different radish cultivars grown in spring and autumn 2020. Raphasatin concentration expressed as phenyl isothiocyanate equivalent concentration. Different capital letters above the error bars indicate significant differences among cultivars within the same season, according to Tukey’s HSD test (*p* < 0.05). Asterisks (**) and ns inside the bars indicate non-significantly difference or significant differences between spring and autumn within the same cultivar according to an unpaired *t*-test (*p* > 0.05, *p* < 0.01, respectively); (**B**) Two-way ANOVA for raphasatin concentrations in radish root among three different cultivars across two seasons. S × C indicates season and cultivar interaction. % in *y* axis of (B) indicates % of explained variation.

In this study, we focused on raphasatin, which is a pungent compound that is present in various radishes [36]. We only quantified raphasatin because other glucosinolate hydrolysis compounds were not detected. Previous studies have reported that neoglucobrassicin or hydrolysis products of neoglucobrassicin are related to the bitter taste of broccoli and cabbage [36,37], but we were not able to detect it. Raphasatin content in the roots of the three cultivars was significantly different between ‘Daeyang 1ho’ and ‘250704’ (*p* < 0.05; Figure 3A). The average of the raphasatin content in the three cultivar radish roots in the spring and autumn was 204.2 and 131.8 μg/g DW (phenyl isothiocyanate equivalent concentration), respectively, being about 1.5 times higher in spring. The raphasatin concentration in spring radish roots of the ‘250704’ cultivar was significantly higher than that in the ‘452949’ cultivar (*p* < 0.05). As determined via two-way ANOVA for season and cultivar, changes in the roots’ raphasatin concentration were also affected by the season (87%, *p* < 0.01, F = 45.79; Figure 3B). The proportions of glucoraphasatin and raphasatin in radish roots differed between cultivars and seasons, which we considered to be caused by a difference in myrosinase activity. Glucosinolate forms a hydrolyzed product via a reaction catalyzed by myrosinase, the activity of which is suppressed at low temperatures and in dry conditions [38,39,40]. Charron et al. [19] reported that myrosinase activity at a 22 °C root temperature was 45.3 U/g and was 1.2 times higher than at a root temperature of 12 °C. Taken together, the high soil temperature in spring, of about 20 °C, might have led to increased myrosinase activity, whereas the low soil temperature in autumn, of about 12 °C, might have suppressed the enzyme activity in this study. Contrary to glucosinolate content, raphasatin content was higher in spring than in autumn.

### 2.4. Water-Soluble Primary Metabolite Changes with Season

**Figure 4:**
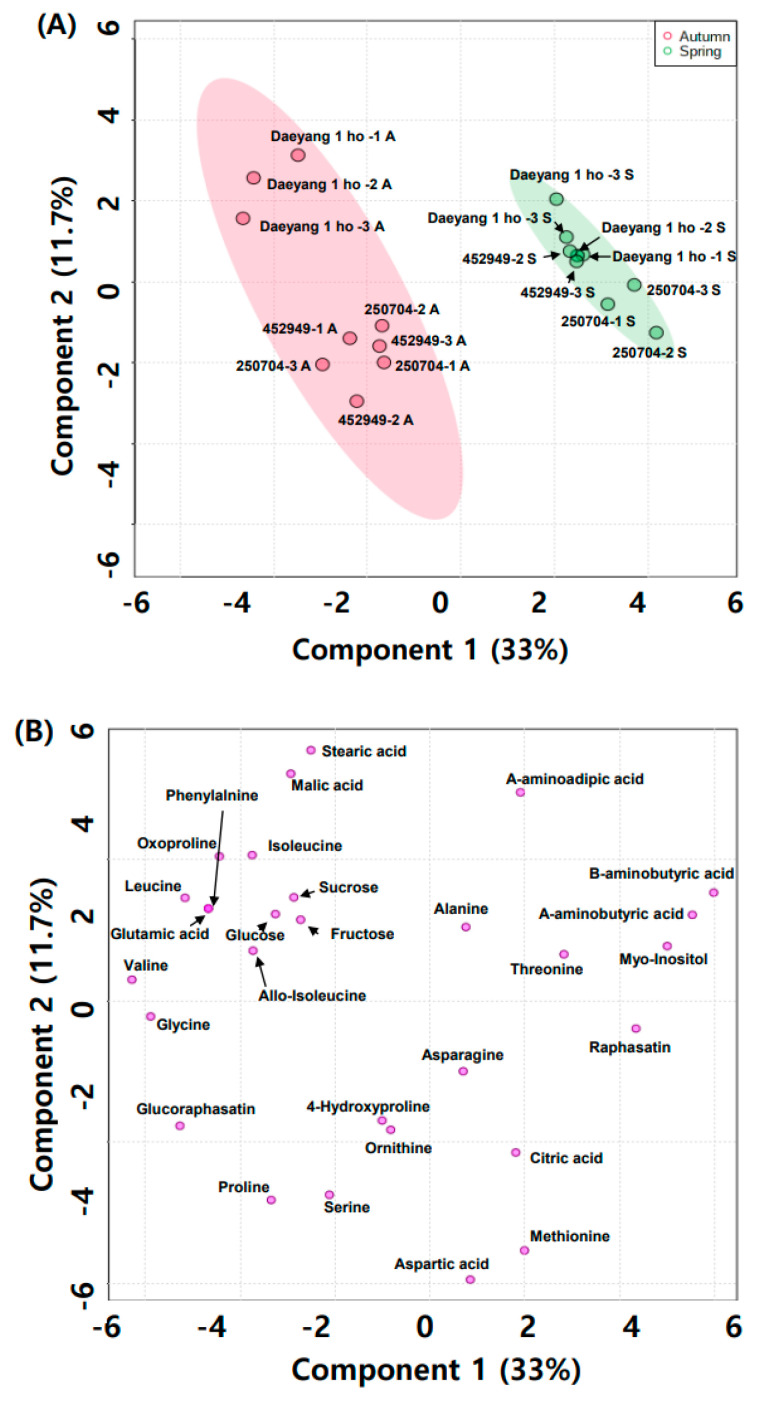
(**A**) Partial least squares discriminant analysis (PLS-DA) score plot; and (**B**) loading plot derived from GC-MS season and cultivar data.

**Figure 5:**
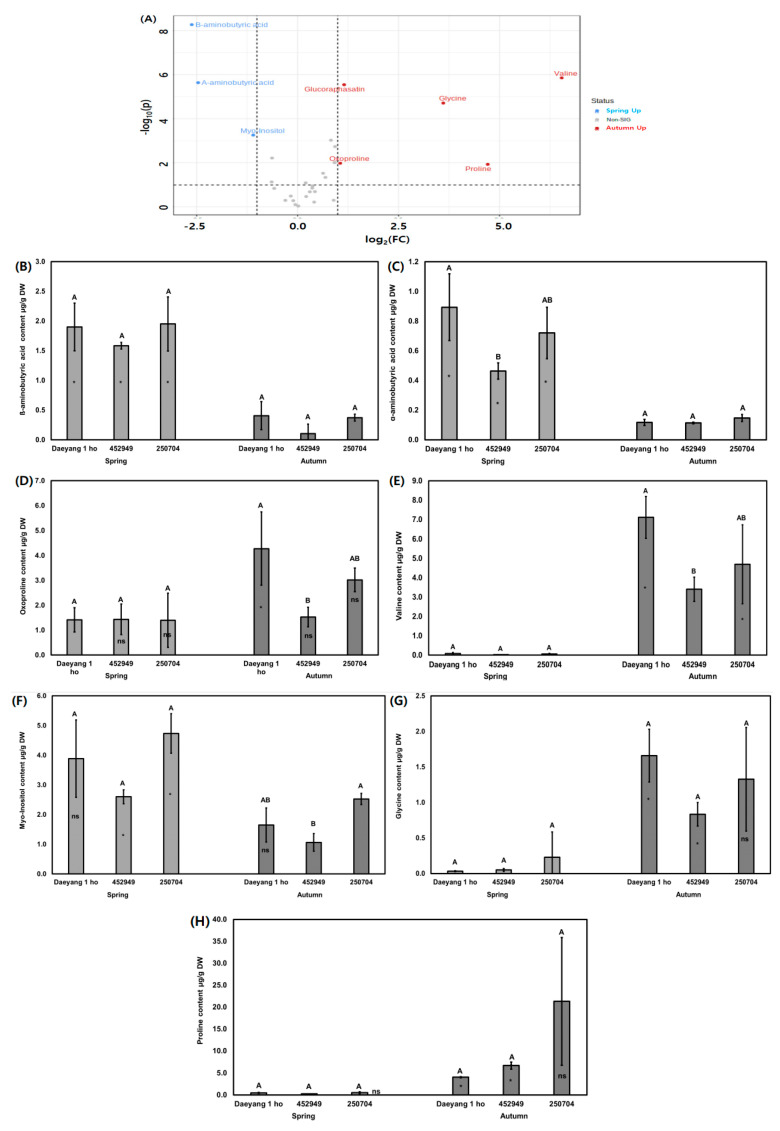
(**A**) Red dots (autumn up) and blue dots (spring up) in the volcano plot indicate significantly different water-soluble metabolites among the different cultivars and seasons (*p* < 0.05); (**B**–**H**) The significantly different contents of water-soluble metabolites amongst the different cultivars and seasons are represented in a bar graph. Metabolite concentrations are expressed as ribitol equivalent concentration. The bars and error bars represent the mean ± standard deviation of three biological replications. Letters above the bars represent significant differences among different cultivars in the same season. Asterisks (*) and ns in the bars represent significant differences among different seasons in the same cultivar, as per Tukey’s HSD test (*p* < 0.05).

We analyzed the primary metabolite water-soluble compounds and amino acids from radish roots using gas chromatograph mass spectrometry (GC-MS). Figure 4A,B presents the clustering patterns obtained using partial least squares discriminant analysis (PLS-DA) score plots and loading plots of different cultivars of radish and different seasons. PLS-DA components 1 and 2 explained 32% and 10.9% of the total variance of the primary radish metabolites from different cultivars and seasons, respectively (Figure 4A). The eight primary metabolites were significantly different, as shown in the volcano plot in Figure 5A. The contents of primary metabolites valine, glycine, proline, oxoproline, and glucoraphasatin in autumn were significantly higher than those in spring. The primary metabolites ß-aminobutyric acid, α-aminobutyric acid, and myo-inositol in spring were significantly higher in content compared to their content in autumn. The relative contents of water-soluble metabolites, displayed as ribitol-equivalent content, are expressed in a bar graph in Figure 5B–H. Proline was known to accumulate in canola and radish under drought stress [41,42,43]. Proline, which is found in high content levels in autumn, was affected by dry soil moisture and rainfall during two weeks before harvesting. In addition, amino acids such as threonine, serine, and proline are known to contribute to sweetness [44,45]. Additionally, compounds of valine and leucine are known to contribute bitterness in the inbred cabbage lines [46]. It was hypothesized that taste and flavor from *Brassicaceae* are affected by amino acids [36]. Several studies reported that free sugars such as glucose, fructose, and sucrose are offset by the bitterness produced by glucosinolate and glucosinolate hydrolysis [47,48,49,50]. We hypothesized that sugar content masked a considerable amount of bitterness from glucosinolate and glucosinolate hydrolysis’ products. Therefore, we subsequently evaluated the absolute sugar content.

### 2.5. Sweetness Value Based on Major Sugar Contents

**Figure 6:**
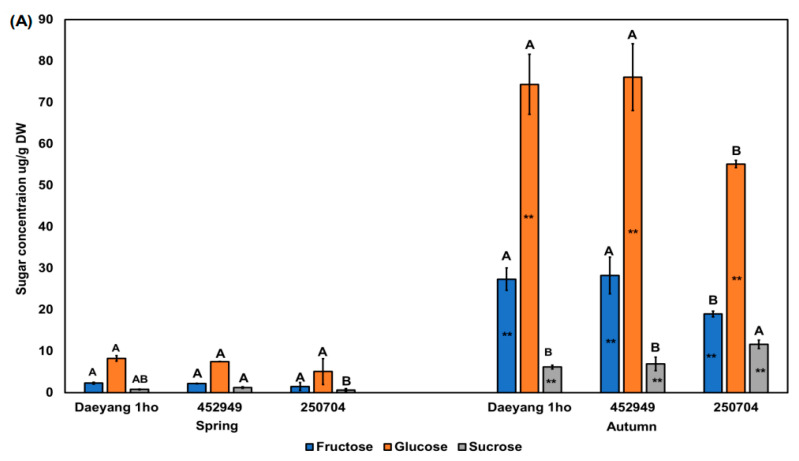

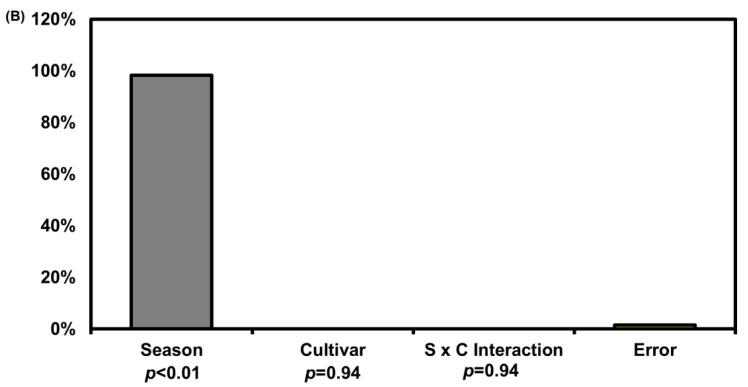
(**A**) Fructose, glucose, and sucrose concentrations in the roots of three different radish cultivars in spring and autumn 2020. Different capital letters above the error bars indicate significant differences among cultivars within the same season according to Tukey’s HSD test (*p* < 0.05). Asterisks (**) inside the bars indicate significant differences between spring and autumn within the same cultivar according to an unpaired *t*-test (*p* < 0.05); (**B**) Two-way ANOVA for raphasatin concentration in radish roots from three different cultivars across two seasons. S × C indicates season and cultivar interaction. % indicated % of explained variation.

We analyzed the sugar content in the roots of the three radish cultivars for the presence of fructose, glucose, and sucrose. We performed quantitative and qualitative analyses of sugar, with the ratios of fructose, glucose, and sucrose as the external standard and ribitol as the internal standard. The sucrose content in the root of the ‘452949’ radish cultivar in spring was significantly higher than that in cultivar ‘250704’ (*p* < 0.05; Figure 6A). Fructose and glucose contents were significantly higher in the roots of cultivars ’Daeyang 1ho’ and ‘452949’ compared to ‘250704’. The sucrose concentration in the autumn radish roots of cultivar ‘250704’ was significantly higher than that in other cultivars in the same season (*p* < 0.05). As analyzed via two-way ANOVA for season and cultivar, the sugar content in the roots was significantly affected by season (98%, *p* < 0.01, F = 363.4; Figure 6B). Our study revealed that seasonal differences in the soluble sugar content were larger than the cultivar effect. Radishes of a few different lines are known to have different soluble sugar contents and proportions of soluble sugar [51]. Previous studies have reported small differences in the soluble sugar composition among 82 lines of radishes [51,52]. Amylase activity hydrolyzes starch to create low-molecular-weight products [53,54]. The enzyme amylase exists in various forms, such as ɑ-amylase, ß-amylase, iso-amylase, and limit-dextrinase [55]. Amylase activity in radish is promoted in cool temperatures, whereas in wheat, it is promoted in mild temperatures [56]. In addition, under dry conditions, sucrose was accumulated because sucrose acts as a compatible solute [57,58]. These previous studies suggested that the soluble sugar content in autumn is higher than that in spring because of promotion of amylase activity at low temperatures and drought stress. Considering high sugar content in autumn compared to spring, this may explain the different radish root pungency by season. Previous studies have reported that the bitterness of Brussels sprouts, cauliflower, and broccoli is offset by perceptions of sweetness [48,49,59]. To confirm the above result in radish, we calculated the ratio between raphasatin to sugar to prove the hypothesis.

### 2.6. Ratio between Raphasatin and Total Sweetness Values from Sugars

**Figure 7:**
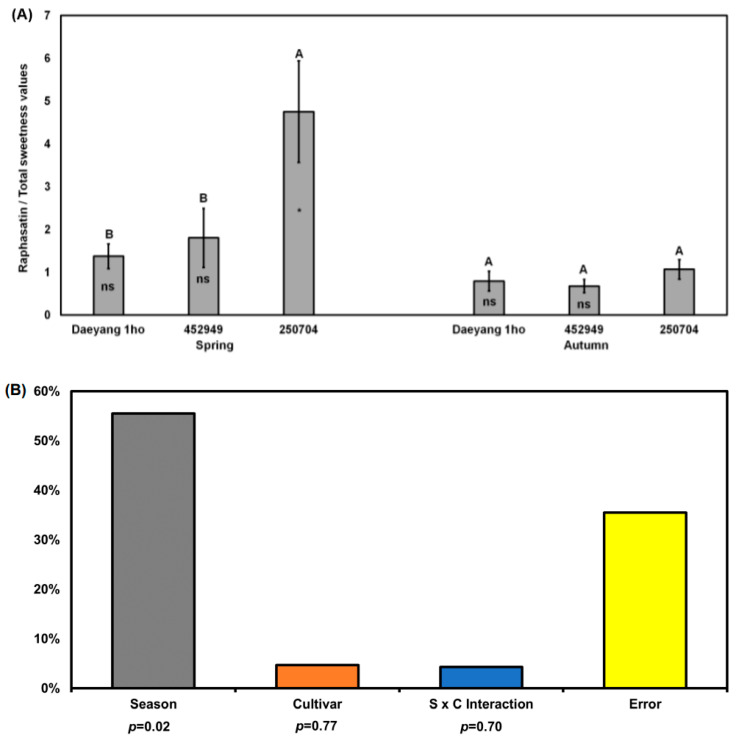
(**A**) Ratio of raphasatin concentration to total sugar concentration in the roots of three different radish cultivars grown in spring and autumn 2020. Total sweetness values from sugars was calculated as follows: (fructose content × 2.0) + (glucose content × 0.6) + (sucrose content × 1.0) based on sweetness value of each sugar [60]. Different capital letters above the error bars indicate significant differences among cultivars within the same season according to Tukey’s HSD test (*p* < 0.05). Ns and asterisk (*) insides the bars indicate non-significantly difference and significant differences between spring and autumn roots within the same cultivar according to an unpaired *t*-test (*p* < 0.05); (**B**) Two-way ANOVA for raphasatin concentration in radish roots from three different cultivars across two seasons. S × C indicates season and cultivar interaction. % indicated % of explained variation.

We analyzed the raphasatin/total sweetness values from sugars ratio that offsets the bitterness in the roots of three radish cultivars to test generally accepted ‘sweetness masking effect on bitter/pungency’ [59]. Total sweetness values from sugars was calculated from the sweetness value of fructose (2.0), glucose (0.6), and sucrose (1.0) to the human mouth [60]. The raphasatin/total sweetness values from sugars ratio in the spring root of cultivar ‘250704’ was significantly higher than that in other cultivars (*p* < 0.05; Figure 7A). In ‘250704’, the ratio of raphasatin/total sweetness values from sugars in the spring roots was significantly higher than that in autumn roots (*p* < 0.05; Figure 7A). As determined via two-way ANOVA, the raphasatin/total sweetness values from sugars in the root was significantly affected by the season (56%, *p* = 0.02, F = 9.566; Figure 7B). The level of pungency was related to the raphasatin/total sweetness values from sugars ratio. Other compounds could be related with radish taste. To our best knowledge, this is the first attempt to explain seasonally changed pungency of radish root. Martyna et al. [59] reported that phenolic compounds in Brassica vegetables(cauliflower and kohlrabi) were not correlated with taste. Thus, this study did not analyze phenolic compounds. A previous study identified bitter-taste-related metabolites using a metabolomic approach with multivariate analysis [37]. As the bitter taste or pungent flavor is determined by many different metabolites and combinations, we successfully employed the metabolomic approach. Based on the results of a previous and the current study, radishes with reduced pungency can theoretically be produced by controlling environmental factors including air temperature, rain fall, and other factors. Practically, we recommend selecting tolerant Altari radish cultivars with high total sugar content for Chonggak kimchi production during late spring and summer. In the Chonggak kimchi production process using small-type radishes, seasonal variations in taste- and flavor-related metabolites in small-type radish are not fully considered. To produce uniform kimchi products, the characteristics of the raw vegetable material in each season should be further explored and investigated.

**Figure 8:**
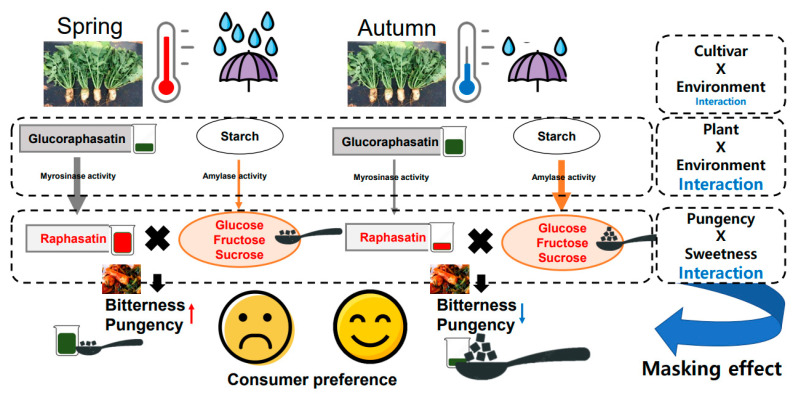
Proposed mechanism of bitterness change of Altari radish by seasonal effect. Significantly lower growing temperature and rainfall before harvest affect glucoraphasatin biosynthesis and myrosinase-mediated conversion and starch-sucrose metabolism. These enzymes and metabolites change by seasonal effect and change the ratio between bitterness/pungency and sweetness. Autumn Altari radish root is less bitter and pungent to consumers due to the masking effect from sweetness of sugars.

In summary, Figure 8 shows the proposed mechanism of bitterness change by seasonal effect. While seasonal effects were significantly different, cultivar effects were not significant on glucoraphasatin and raphasatin (*p* = 0.29 and *p* = 0.97). Myrosinase and amylase activities might have been changed by different environmental factors including temperature and soil moisture [38,39,40] which require further experiment to confirm, but previous studies have reported these enzyme activities were changed by environmental conditions [19,38,39,40]. Principles of the bitterness of radish have been identified by previous studies [61,62]. However, seasonally changed raphasatin does not fully explain the bitterness change of radish root between spring and autumn. Recent studies also imply that sweetness of sugars can mask bitterness or pungency of brassica vegetables [59] (Figure 8). Our new proposed mechanism to explain bitterness of Altari radish by seasonal effect is a ratio between raphasatin and total sweetness values from sugars. This proposed mechanism of the bitterness change of Altari radish by seasonal effect may be helpful to improve consumers’ preference and reduce consumers’ complaints on bitter/pungent Altari Kimchi.

## 3. Materials and Methods

### 3.1. Cultivation and Growing Conditions

In this study, we cultivated radish during spring and autumn following commercial practices. Three small-type radish cultivars were selected based on their level of pungency, for spring cultivation (after personal communication with breeders): mild (‘Daeyang 1ho’), moderate (‘452949’), and strong (‘250704’). Both spring and autumn cultivations were conducted at the same location—Incheon, Gyeonggi-do, Korea (37°07′58.5″ N, 127°37′41.6″ E)—from April to June 2020 for spring planting and September to November 2020 for autumn planting. The soil was fertilized with 10.4 kg of urea, 54.4 kg of fused phosphate, 7.2 kg of potassium chloride, 60 kg of calcium hydroxide, 1.2 kg of borax, and 160 kg of sawdust compost per 0.1 ha. Two seeds were directly sown in holes in a firm, mulched field on 29 April 2020 for spring cultivation and 15 September 2020 for autumn cultivation in two rows (35 cm apart) per bed, with a spacing of 25 cm between plants within the row.

We regularly measured air and soil temperature and soil moisture from seed sowing to harvest in both spring and autumn plantings. We measured air temperature at a height of 60 cm above the ground. We determined soil temperature 15 cm below the mulch from the soil surface. Air and soil temperature were measured with an Ondotori thermos recorder (TR-71Ui, T&D Corp., Nagano, Japan) in 30-min intervals over the growing season. Soil moisture was measured using a TEROS 10 (Meter Group Devices, Pullman, WA, USA), a soil moisture sensor, installed 20 cm under the soil surface near the rootzone. Rainfall data were obtained from the Korea Meteorological Administration.

Radishes were harvested on 21 June 2020 and 17 November 2020 for spring and autumn plantings, respectively. The leaf and root samples were stored in a cryogenic freezer immediately after harvest and lyophilized for metabolite analysis.

### 3.2. Quantification of Glucosinolate

Glucosinolates were analyzed following the methods described in a previous study [13,63]. First, 0.1 g of freeze-dried radish was extracted with 2 mL methanol (70%) at 95 °C for 10 min; after cooling the extract for 5 min, sinigrin was added to it as an internal standard. After vortexing, we centrifuged the sample at 13,475× *g* for 10 min at room temperature (20 °C~22 °C). After collecting the supernatants, pellets were extracted with 2 mL methanol (70%). A 15 mL mixture of 1 M lead acetate and 1 M barium acetate (1:1, *v*/*v*) were added to 1 mL of pooled extract, which was centrifuged at 12,000× *g* for 1 min after vortexing for protein precipitation. We poured DEAE Sephadex A-25 resin (1 mL, 1:1 (*v*/*v*) in water; GE Healthcare, Piscataway, NJ, USA) into poly-prep columns. We poured 3 mL of 0.02 M pyridine acetate and pooled extract into the pre-charged column. We poured 3 mL of deionized distilled water into the column. After passing the deionized distilled water through the resin, we added 500 μL of sulfatase solution (20 U/mL). The column was incubated overnight at room temperature (20 °C~22 °C). After passing 3 mL of deionized distilled water, passed solution was collected. The collected sample was filtered using a 0.22 μm nylon syringe filter; 1 μL of sample was analyzed using an Agilent 1100 series HPLC (Agilent Technologies, Santa Clara, CA, USA) equipped with a photodiode array detector. We used a Ascentis 50813-U C18 (10 cm × 2.1 mm i.d., 2 μm). Deionized distilled water (mobile phase A) and 100% acetonitrile (mobile phase B) were used with the following gradient conditions: 0 min 0.5% B, 1 min 0.5% B, 5 min 5% B, 9 min 40% B, 11 min 40% B, 12 min 0.5% B, and 15 min 0.5% B, with a flow rate of 0.35 mL/min. The oven temperature was set at 40 °C. Glucosinolates were identified by measuring absorbance at 229 nm, followed by comparison of the noted absorbance with the results of a previous study [64]; subsequently, glucosinolate content was quantified using the internal standard (sinigrin) and relative response factor [65].

### 3.3. Glucosinolate Hydrolysis Products Measurement

The glucosinolate hydrolysis products were analyzed using GC-MS. For this purpose, 50 mg of freeze-dried radish powder was extracted with 1.0 mL of deionized distilled water. The sample was incubated for 10 min at room temperature, and the sample extract was centrifuged for 2 min at 12,000 × *g*. We transferred the collected supernatant (0.5 mL) to a Teflon tube (Savillex Corporation, Eden Prairie, MN, USA); we added 490 μL of dichloromethane to the Teflon tube; 10 μL of phenethyl isothiocyanate was then added as the internal standard. The sample extract was incubated for 6 h at 40 °C with the endogenous enzyme myrosinase to hydrolyze the glucosinolates. We centrifuged the incubated sample at 12,000× *g* for 2 min. The lower organic layer was collected in a GC vial.

For the analysis of the glucosinolate hydrolysis products, we used a GC device (GCMS-QP2020 NX, Shimadzu, Japan) coupled to an MS detector system (Nexis QC-2030, Shimadzu, Japan) and an autosampler (AOC-20i/s plus, Shimadzu, Japan). We analyzed the products using a capillary column (DB-5MS, Agilent Technologies, Santa Clara, CA, USA; 30 m × 0.25 mm × 0.25 μm). The oven temperature was maintained at 35 °C for 1 min, increased at a rate of 40 °C/min to 310 °C, and then maintained for 5 min. We set inlet and ion source temperatures to 270 and 300 °C, respectively. We obtained the mass spectra in electron ionization mode with a scan range of *m*/*z* 40–450, and we set the flow rate of the helium carrier gas at 1.2 mL/min. We injected 1 μL of the sample at a split ratio of 4:1. The glucosinolate hydrolysis products were quantified as the relative ratio of phenyl isothiocyanate.

### 3.4. Water-Soluble Primary Metabolite and Sugar Contents

We analyzed the water-soluble primary metabolites from the aqueous layer; we transferred 50 µL of the upper layer to 1.5-mL microcentrifuge tubes. After adding 5 µL of 10 mg/mL ribitol as an internal standard, the 1.5 mL tubes were dried in a speed vacuum (Vision, Bucheon, Gyeonggi-do, Korea) at room temperature for at least 6 h. To the dried samples, we added 50 μL of a freshly prepared mixture of 40 mg/mL methoxyamide in pyridine and incubated at 800× *g* for 90 min at 37 °C. Then, we added 80 μL of *N*-methyl-*N*-(trimethylsilyl)trifluoroacetamide (MSTFA) to the incubated sample, which was incubated at 800× *g* for 20 min at 50 °C. The oven temperature was maintained at 80 °C for 1 min, increased at a rate of 15 °C/min to 330 °C, and then maintained for 5 min. The injector and detector temperatures were set at 205 and 250 °C, respectively. We obtained the mass spectra in electron ionization mode with a scan range of *m*/*z* 40–550, and we operated the spectrometer in positive electron impact mode at an ionization energy of 70.0 eV [66]. Then, 1 μL of the sample was injected at a split ratio of 300:1, and the flow rate of the helium carrier gas was set at 1.2 mL/min. We identified the major water-soluble primary metabolites using the National Institute of Standards and Technology (NIST) library and standard compounds for the metabolomic study. The contents of major sugars, including fructose, glucose, and sucrose, were quantitated using external standards.

### 3.5. Raphasatin Content/Total Sweetness Value from Sugars Ratio

We calculated the ratio of the raphasatin content to total sweetness value from sugars, to quantify the bitterness. Raphasatin content was quantified as the relative ratio of phenyl isothiocyanate. To estimate the sweetness values, total sugar content was recalculated as follows: (fructose content × 2.0) + (glucose content × 0.6) + (sucrose content × 1.0) [60].

### 3.6. Statistical Analysis

We determined the significance of all the experimental results using Prism 5 (GraphPad, Northside, San Diego, CA, USA). To test for any standard differences between experimental treatments, we applied ANOVA. Once significance was identified, the similarity among the treatments was estimated using Tukey’s HSD test. Any significance for values of means was tested within a confidence level of *p* < 0.05. Soil moisture, soil temperature, rainfall, and air temperature (mean, minimum, and maximum values) during spring and autumn plantings were compared by independent sample *t*-test in SPSS (version 12.0 for Windows; SPSS, Chicago, IL, USA).

## 4. Conclusions

Small-type radish grown in spring is known to be more bitter than autumn-grown radish. In contrast to the increased glucoraphasatin concentration, the bitterness compound, raphasatin, is present in lower concentrations in autumn-grown radish, compared to spring-grown radish. As a result of analyzing polar metabolites, in this study, we found that seven amino acids, glucoraphasatin, and sugars in three different radish cultivars are significantly different between spring- and autumn-grown radishes. These significantly different amino acids can be used as biomarkers to differentiate the radishes grown in these seasons. We found that the ratio of raphasatin content to total sweetness value from sugars is related to the pungency of the three small-type radish cultivars grown in two different seasons. Our experimental results suggested that the pungency of small-type radishes is mainly affected by the ratio of raphasatin content to total sweetness value from sugars, wherein the sweetness can mask the bitter taste and pungent flavor. Although the seasonal effect is considerably strong, cultivar selection is still an important factor in avoiding seasonal-associated pungency. Based on the levels of sensorial-related metabolites in the different seasons, Altari (Chonggak) radish kimchi could be modified in its recipe to avoid consumer complaints.

## Data Availability

All data generated or analyzed during this study are included in this published article.

## Supplementary Materials

The following supporting information can be downloaded at: https://www.mdpi.com/article/10.3390/plants11030312/s1, Figure S1: Chromatogram of glucosinolate from small-type radish root by HPLC. Table S1: Three glucosinolate concentration of root of three different radish cultivars grown in spring and autumn 2020.
