# Supplementary material for: Seasonal Effects of Glucosinolate and Sugar Content Determine the Pungency of Small-Type (Altari) Radishes (Raphanus sativus L.)
Source: Plants (Basel). 2022 Jan 25;11(3):312. doi: 10.3390/plants11030312 (PMC8839410; doi:10.3390/plants11030312)
Supplement: Supplementary file 1 [file plants-11-00312-s001.zip › plants-1544221-supplementary.pptx]

## Slide 1
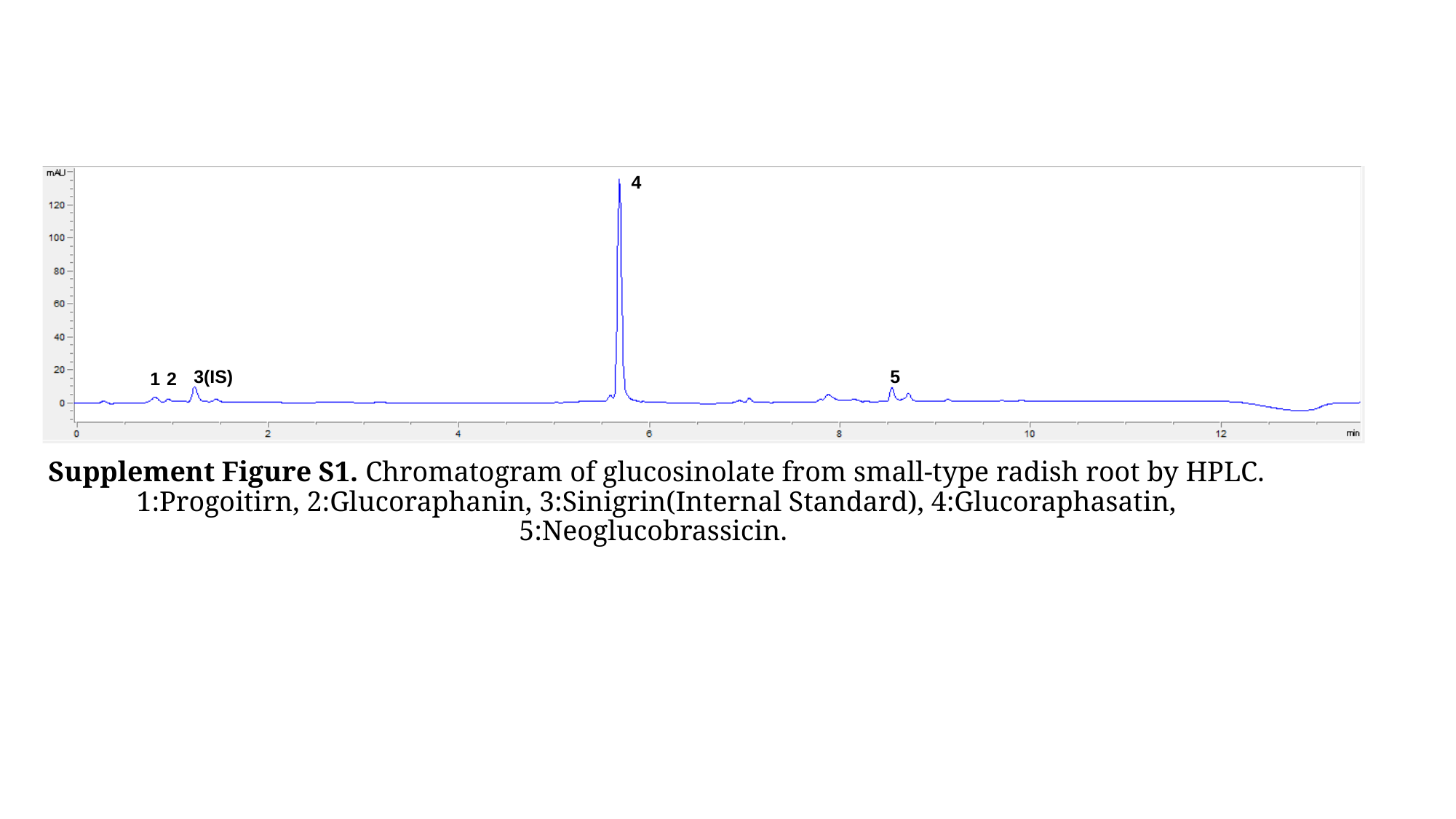

4
5
3(IS)
1
2
Supplement Figure S1. Chromatogram of glucosinolate from small-type radish root by HPLC. 1:Progoitirn, 2:Glucoraphanin, 3:Sinigrin(Internal Standard), 4:Glucoraphasatin, 5:Neoglucobrassicin.

## Slide 2
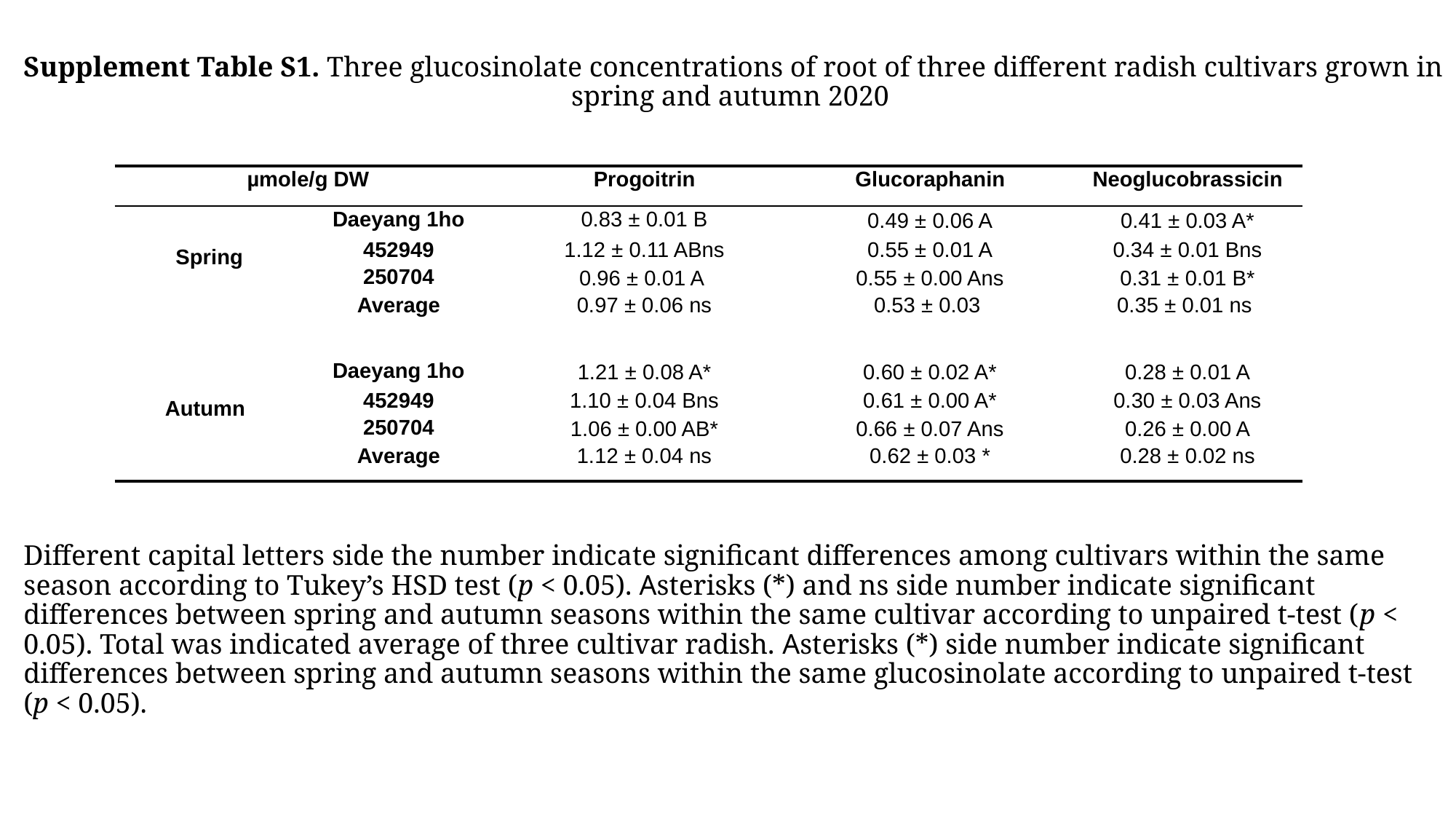

Supplement Table S1. Three glucosinolate concentrations of root of three different radish cultivars grown in spring and autumn 2020
| µmole/g DW | | Progoitrin | Glucoraphanin | Neoglucobrassicin |
| --- | --- | --- | --- | --- |
| Spring | Daeyang 1ho | 0.83 ± 0.01 B | 0.49 ± 0.06 A | 0.41 ± 0.03 A\* |
| | 452949 | 1.12 ± 0.11 ABns | 0.55 ± 0.01 A | 0.34 ± 0.01 Bns |
| | 250704 | 0.96 ± 0.01 A | 0.55 ± 0.00 Ans | 0.31 ± 0.01 B\* |
| | Average | 0.97 ± 0.06 ns | 0.53 ± 0.03 | 0.35 ± 0.01 ns |
| | | | | |
| Autumn | Daeyang 1ho | 1.21 ± 0.08 A\* | 0.60 ± 0.02 A\* | 0.28 ± 0.01 A |
| | 452949 | 1.10 ± 0.04 Bns | 0.61 ± 0.00 A\* | 0.30 ± 0.03 Ans |
| | 250704 | 1.06 ± 0.00 AB\* | 0.66 ± 0.07 Ans | 0.26 ± 0.00 A |
| | Average | 1.12 ± 0.04 ns | 0.62 ± 0.03 \* | 0.28 ± 0.02 ns |
Different capital letters side the number indicate significant differences among cultivars within the same season according to Tukey’s HSD test (p < 0.05). Asterisks (*) and ns side number indicate significant differences between spring and autumn seasons within the same cultivar according to unpaired t-test (p < 0.05). Total was indicated average of three cultivar radish. Asterisks (*) side number indicate significant differences between spring and autumn seasons within the same glucosinolate according to unpaired t-test (p < 0.05).
